# An Analysis of the Relationship Between Staff Qualification and Export Readiness of Pharmaceutical Companies: The Case of Iran

**Published:** 2012

**Authors:** Mehdi Mohammadzadeh

**Affiliations:** *School of Pharmacy, Shahid Beheshti University of Medical Sciences, Tehran, Iran. *

**Keywords:** International marketing, Pharmaceutical marketing, Export readiness, Internationalization, Staff qualification, Iran

## Abstract

Export and the readiness to export constitute the first step of international marketing, which are affected by both internal and external factors of firms. One of the most important internal factors is the presence of skilled personnel. The purpose of this study was to define the relationship between staff qualification and encouragment with the readiness level of Iranian pharmacuetical firms for engagement in export marketing.

The research was based on a single case study on a basket of seven leading domestic firms. For the bias reduction, questionnaires as well as interviews with managers were used.

The performance of the studied factor was lower than the desired level for export readiness and there was much scope for improvement in staff qualifications to achieve such readiness.

The results of this research enable small and medium-sized pharmaceutical companies to evaluate their staff qualification levels needed for export readiness and to detect their shortcomings in order to improve them.

## Introduction

From 1979, the attention of pharmaceutical industries and the healthcare system of Iran has been progressively diverted toward ethical concerns. This stance was initially adopted as a reaction to monopolies of large multinational pharmaceutical enterprises and entailed extensive growth of investments in that industry.

In the past three decades, large investments, mainly by public and private sectors have been made in pharmaceutical industries in Iran, and several small and medium-sized companies have been established in different parts of the country.

Despite of this investment and increasing the production capacity, field of activity of Iranian pharmaceutical companies have been limited to domestic market and according to MOH (Iran Ministry of Health) reports, the Iranian pharmaceutical companies export only 5% of their products (1).

The export performance of companies has been studied widely, and the literature reports factors affecting export performance that are both internal and external to company. The former factors include marketing strategy, organizational structure, managerial experience (staff qualification), and/or resource availability (2). External factors such as industry competitiveness, business environment, or product characteristics are also linked to export performance (3, 4).

A prerequisite for moving toward the international markets is the readiness of companies in terms of both internal and external affecting factors (5). However, in the Iranian pharmaceutical companies, no systematic scientific study has been carried out to evaluate the readiness situation or performance of the pharmaceutical companies for export.

According to Bishop’s study in the United State Agency for International Development (USAID) report, various quantitative and qualitative factors should be considered when determining export readiness. Some of these elements are summarized as follow: Exporting experience, Health of company, Production capacity, Product quality, Certifications or applicable registrations, speed to market and qualified human resources (6). 

Different models and assessment methods have been developed with regard to export readiness. 

To understand the competitive advantage of nations in international business and export activities, Michael Porter offers a model called the “Diamond Model.” This model can explain the competitive position of a nation in the global context (7).

The first factor consists of firm strategy, structure, and rivalry and includes internal factors such as management and leadership style, qualified staff, efficient resources, and effective strategies in different areas (production, distribution, and marketing) are prerequisite for initiating of export marketing (8).

Rahman has introduced a Model (Rahman Model) that is about an effective international positioning process. This model specifically obtains the type of information “international business decision makers” consider for appraising and tailoring a customized international positioning process and identifies different pieces of information essential and appropriate for the proposed positioning process (9).

Sharma *et al, *have used multiple criteria decision analysis methods for utilizing the information obtained in the study and shaping up international positioning process (10).

The nature of pharmaceutical marketing is significantly different from other types of marketing such as consumer marketing. This is largely due to the differences in the nature of pharmaceutical products and the industrial atmosphere of pharmaceutical firms, compared with other consumer goods due to operational differences in the healthcare environment. Hence, from a theoretical point of view, we can consider the pharmaceutical business as a subset or slice of marketing science based on some general shared principles as well as specific individual inter-industry considerations (11). 

From the customer point of view, pharmaceutical marketing is a multi-type customer criterion, as the final consumers of the products are individual people, and they may expect products similar to consumer products and core marketing principles applicable to them. However, pharmaceutical marketing has specific unique characteristics, because the decision makers for buying are not the consumers. Definitely other individuals or organizations such as physicians, hospitals, governors, etc have been involved (12). Thus, the presence of qualified staff for creating effective relationship is one of the most important success key factors.

The first important differentiating factor in this industry is the tough regulatory and control systems. This is due to the need for careful delivery of products to patients who are in need for care and its association with human safety and life. Therefore, managing of regulatory affair is a knowledge base activity that should be done by expert staff (13). 

Export procedures for the two defined classes of medicines including: over the counter (OTC) and prescriptive are considerably different. Registration and licenses from the healthcare system authorities are relatively easy for OTC medicines. However, they are more cumbersome for prescription-based ones. Sometimes export involves just for a single prescription outside the normal fashion. Although after passing of regulatory process, mechanism of marketing is similar to consumer goods, but in all stages of market management from registration to dispensing, direct encountering of customer and staff is desperately needed. Even in case of E-pharmacies (Internet-based pharmacies) the delivery of medicines to patients by mail and without direct face-to-face relation between pharmacist and patients is not permitted by many of health care systems. An example of this situation is the delivery of medicines by Canadian E-pharmacies (Internet-based pharmacies) against prescriptions issued by US physicians. This business came up with the criticism by both prescription regulations and ethical norms of healthcare systems (14). 

Awareness of pharmacists working in drugstores about counterfeit drugs is necessary for healthy supplying of medicines to patients specially for delivery to a patients by mail and without direct face to face relationship between pharmacists and patients. Pharmacists should have good qualification and knowledge regarding counterfeit drugs (15).

## Experimental

Our research was mainly qualitative, since the aim was to gain a profound understanding of how staff qualification and encouragement could affect export readiness in pharmaceutical companies and the situation in sample firms. Our sample consisted of seven leading Iranian pharmaceutical firms. We did not analyze the companies individually, but the data were gathered globally. Therefore, our strategy followed a holistic single case design, combining multiple units of seven embedded firms.

The top seven companies in the industry were selected for this purpose. The inclusion criteria were according to MOH reports about their activities. Their total shares were 40% and 70% of the domestic market and pharmaceuticals exports, respectively. 

We used both questionnaire and interview to gather information from the key persons and management teams of the firms (several rounds of interviews had been carried out with some individuals). The reason for such approach of the dual data gathering system in this phase was to reduce deviations and bias and to increase as much as possible the validity and reliability of the research by triangulation. At least ten experts in each of the firms were interviewed or filled the questionnaires.

We examined the content validity by an evaluation questionnaire. We asked the experts to evaluate relevance, completeness and clarity and then the scores of each question were defined. To test the reliability of the questionnaire, we determined the Cronbach’s Alpha Coefficient for each one. Finally, after some exclusion, a list of five questions (Cronbach coefficient more than o.7) was obtained through this process. Each question represents an element.

The final questionnaire contained multiple questions about each of the elements. We asked the responders to score from 1 to 6 for each element based on the importance and performance. 

## Results

After finalizing the elements of factor through defining of the alpha Cronbach coefficient, we were able to evaluate performance and importance of each elements for analyzing the gap.

*Performance of the factor*

The statistical hypothesis was defined as following:

H_0_: μ≤ 3

H_1_: μ≤ 3

Hypothesis *H*_1_ explains that the average of the scores of performances shall be greater than 3, with an accuracy of 95%, and the *H*_0_ hypothesis shows that the average should be less or equal to 3. This hypothesis shows a weakness of the firm’s performance in this dimension. The results showed that the *t *score for performance of staff qualification and encouragement was 11.034 with an average of 4.11 and SD of ±0.870 the degree freedom was 73. Hence, according to 

*t*_0_ = 11/034 > *t*_0/05,73 _= 1/645

and accuracy level (p = 0.000 < 0.05), *H*_0_ was rejected and *H*_1_ showed improvable performance of the sample firms in relation to staff qualification and encouragement to export and international marketing in sample firms.

*Importance of the factor*

Statistical hypotheses *H*_0_ and *H*_1_ were defined as the following: 

H_0_: μ≤ 3

H_1_: μ≤ 3

*t *score for importance of staff qualification and encouragement was 39.149 with an average of 5.29 and SD of ±0.504 the degree of freedom was 73.

Thus, according to:

*t*_0_ = 39.149 > *t*_0/05,73_ = 1/645 

and accuracy level (p = 0.000 < 0.05), *H*_0_ was rejected and *H*_1_ shows high importance of staff qualification and encouragement in relation to export and international marketing in the sample firms.

*Ranking and comparisons of performances and importance of elements*

As it has shown in table 1, the first rank for performance of sample firms with regard to evaluated elements was belonged to the proudness for working in the company (with an average performance of 4.67). On the other hand, the lowest rank contained the constant training (with an average performance of 3.24). These indicated that despite being successful in creating a pleasant atmosphere for staff to be proud of working in such companies, there was a significant weakness in the training process continuously in the firms studied.

**Table 1:** Comparison of the performance of sample firms in each element

**Element**	**Rank**	**Average Rank**	**Average Performance**
Proudness for working in the company	1	3.73	4.67
Staff enthusiasm toward exporting	2	3.11	4.33
Management Turnover *	3	3.09	4.18
Existence of an “export champion”**	4	3.03	4.03
Training constantly	5	2.05	3.24

Notes: * Turnover of each manager in his/her position is more than five years. ** An expert senior manager who motivate for exporting

Nonetheless, for ranking of elements regarding importance, the responses to the questionnaire involved in an anticipated glimpse, and showed different results. Table 2 shows that the existence of an expert manager who drives the exporting successfully, so called “an export champion”, was assigned as the first rank. Staff enthusiasm for export consisted the lowest rank. The other elements laid between the extremes.

**Table 2:** Comparison of the importance of sample firms in each element

**Element**	**Rank**	**Average Rank**	**Average Importance**
**”Existence of an “export champion	1	3.47	5.6
Training constantly	2	3.06	5.4
Proudness for working in the company	3	3.16	5.37
* Management Turnover	4	2.66	5.09
Staff enthusiasm toward exporting	5	2.66	5.03

* Turnover of each manager in his/her position is more than five years. ** An expert senior manager who motivate for exporting

**Table 3:** Paired sample T test for gap identification between importance and performance

**Dimension**	**Average**		**t**	**Degree of Freedom**	**p**
Staff qualification and encouragement	Importance	5.29	10.483	73	0.00
	performance	4.11			

*Analyzing the gap between importance and performance*

The gap between importance and performance was determined by paired samples t-test. 

The main aim of this section was to check for any possible significant gap between the importance (expected level) and performance (current condition) of factor’s elements.

For each element, two statistical hypotheses are defined individually as follows:

H_0_: μ_1_ = μ_2 _

H_1_: μ_1_ ≠ μ_2_

Hypothesis *H*_1_ states that the average of importance of the factor is relatively different from the average of performances in the same factor. There was a considerable gap between these two in case of lower averages of performance in comparison to importance; this means that the firms were still considered not to be strong enough in this factor and in behind of desired levels.

The average of importance was 5.29 and the average of performance was 4.11. We have the formulai *t*_0_ = 10.483 > *t*_0/05,73_ = 1/645 and the asymptotic significance was zero (less than 0.05). Overall, the statistical hypothesis *H*_0 _was rejected in favor of hypothesis H_1_ and the gap between importance and performances was attendable. As the average of performance is less than that of importance, the sample firms were weaker than the desired level. 

## Discussion

The relationship between human creativity and staff qualification with export performance have not been studied in depth; and the field of researches have been focused on the product quality and technology. The current study highlights the elements that have been usually ignored in the presence of pharmaceutical firms in the foreign markets. 

As mentioned before, in total five elements for this factor were defined and evaluated, and the importance of those (desired level of firms) considered as well as the their performance (present level of firms) were highlighted. Furthermore, the gap between firms’ performance and the importance of their elements were presented, showing how far the firms are behind the desired level. 

The results showed that all five elements were important for the export readiness of the firms. However, the degree of importance of the mentioned elements varied significantly. The highest rank was given to the presence of an export champion in the company, indicated that the human creativity is one of the most important factors for export success.

As the results showed, all the elements are significantly important. Indeed, the knowledgeable, qualified, and well-motivated staff are required for successful implementation of export marketing plans. Among the other elements, having an export champion in the company, continuous training in export marketing, continuity of managers working in their position, and staff being enthusiastic toward exporting and presence in international market are worth mentioning. The results are summarized in the diagram below. Lack of continuous training in export marketing seems the largest weak point of firms. Furthermore, lack of a senior manager and an export champion in the company is the next distinguished weakness, followed by the lack of stability of management and a weak interest of staff toward exporting, along with the presence in foreign markets. However, staff being proud of working in the company enjoys a better situation.

Already, the export performance of companies has been studied widely as mentioned in literature, and many literatures exist regarding. The evaluation of to evaluating different factors affecting export performance. To the best of our knowledge, the current study is the first one that showed the importance of qualified staff in export marketing. 

## Conclusion

In conclusion, the results show that all elements considered in this factor are performing below the required level for success in an export market. Based on research questions, the five above-mentioned elements are the main aspects of staff qualification for evaluating companies that are willing to export, and Iranian pharmaceutical companies are far behind the desired level. For improving the situation and moving toward acceptable levels, the Iranian pharmaceutical companies can consider some of the following recommendations:

Focus on staff and management training to increase their knowledge, especially in field of marketing and international business. 

Increase the stability of management system and rationalize turnover of managers of firms.

Encourage stakeholders, especially staff to be enthusiastic toward exporting and presence in foreign markets. 
